# Astaxanthin Promotes Nrf2/ARE Signaling to Inhibit HG-Induced Renal Fibrosis in GMCs

**DOI:** 10.3390/md16040117

**Published:** 2018-04-04

**Authors:** Qing Chen, Jun Tao, Xi Xie

**Affiliations:** 1 Hainan Key Laboratory of Sustainable Utilization of Tropical Bioresources, Hainan University, Haikou 570228, China; chenqing1984@hainu.edu.cn (Q.C.); taoj@hainu.edu.cn (J.T.); 2 School of Life Science, Institute of Tropical Agriculture and Forestry, Hainan University, Haikou 570228, China; 3 Key Laboratory of Tropical Biological Resources of Ministry of Education, Hainan University, Haikou 570228, China; 4 Hainan Provincial Key Laboratory for Tropical Hydrobiology and Biotechnology, College of Marine Science, Hainan University, Haikou 570228, China

**Keywords:** astaxanthin, oxidative stress, renal fibrosis, diabetic nephropathy, Nrf2/ARE signaling

## Abstract

Oxidative stress is the main cause of diabetic nephropathy (DN) progression. Nuclear factor-erythroid 2-related factor 2 (Nrf2)/antioxidant response element (ARE) signaling is a crucial cellular defense system to cope with oxidative stress. Astaxanthin (AST) is a fat-soluble xanthophyll carotenoid with remarkable antioxidative capacity. AST exerted renal protective in diabetic rats. This study aimed to determine whether AST could alleviate the pathological progress of DN by activating Nrf2/ARE signaling and diminishing the excessive oxidative stress and fibronectin (FN) accumulation in glomerular mesangial cells (GMCs) challenged with high glucose (HG). In the current study, we found that AST treatment alleviated the metabolic parameters, renal morphology and extracellular matrix (ECM) accumulation in streptozotocin-induced diabetic rats. Additionally, HG induced the adaptively activated Nrf2/ARE signaling and increased the expression of FN, intercellular adhesion molecule-1 (ICAM-1) and transforming growth factor-β1 (TGF-β1), as well as the intracellular reactive oxygen species (ROS) generation in GMCs. However, AST treatment strongly promoted the nuclear translocation and transcriptional activity of Nrf2 as well as upregulated the expression of superoxide dismutase (SOD1), NAD(P)H: quinone oxidoreductase (NQO1) and heme oxygenase-1 (HO-1), ultimately quenching the higher level of ROS and inhibiting the FN, ICAM-1 and TGF-β1 expression induced by HG. Collectively, our data suggest that the renoprotective effect of AST on DN depends on Nrf2/ARE signaling activation, which could be a potentially therapeutic strategy in the treatment of DN.

## 1. Introduction

Diabetic nephropathy (DN) is a severe microvascular complication of diabetes that is characterized by sequential pathology, including renal hypertrophy and basement membrane thickening at the early stages and extracellular matrix (ECM) (such as fibronectin (FN) and collagen accumulation), glomerulosclerosis, and interstitial fibrosis at the latter stages, ultimately leading to renal function loss and end-stage renal diseases [1,2].

Glomerular mesangial cells (GMCs) play important roles in the physiological and pathological processes of kidneys. GMCs generate ECM, intercellular adhesion molecule-1 (ICAM-1) and transforming growth factor-β1 (TGF-β1), which trigger the thickening of the glomerular and tubular basement membranes. This phenomenon accelerates the pathological processes of glomerulosclerosis and tubulointerstitial fibrosis, leading to DN [3,4]. As a key component of ECM, FN is generally used as an index for evaluating the extent of matrix accumulation. Thus, inhibiting FN in GMC production is an effective strategy for ameliorating DN.

Hyperglycemia is the primary pathogenetic factor of diabetic renal diseases. The pathogenesis of DN is associated with glucolipid metabolism disorders, renal hemodynamic changes, and increased non-enzymatic glycation of proteins. Oxidative stress is the main cause of DN progression. An increase in reactive oxygen species (ROS) levels in renal cells cultured under high glucose (HG) conditions, and an excessive accumulation of ROS in kidney tissues, can result in continuous oxidative stress, causing renal damage [5,6]. In addition, ROS can activate cytokines to facilitate the overproduction of FN and ICAM-1, which initiates and participates in the pathogenesis of diabetic renal fibrosis. Moreover, ROS can generate abundant cytokines and growth factors to enhance renal cell proliferation or hypertrophy by upregulating ECM and TGF-β1 expression while decreasing their degradation, leading to diabetic renal fibrosis [7,8]. Thus, reducing excessive ROS is crucial for the prevention and treatment of DN.

Nuclear factor-erythroid 2-related factor 2 (Nrf2) is a redox-regulated transcription factor that determines the expression of a battery of approximately 250 genes involved in various cellular functions [9]. In addition, Nrf2 contributes to the cytoprotection against environmental electrophiles and oxidative stress [10,11]. Upon oxidative and electro-philic insults, Nrf2 dissociates from its cytoplasmic repressor Kelch-like ECH-associated protein 1 (Keap1) and then translocates to the nucleus, where it binds to antioxidant response element (ARE) and regulates the expression and coordinated induction of a battery of genes encoding chemopreventive proteins, including detoxifying enzymes NAD(P)H: quinone oxidoreductases (NQO1 and NQO2), glutathione S-transferase, superoxide dismutase (SOD1 and SOD2) [12], glutamylcysteinyl synthetase, and heme oxygenase-1(HO-1) [13]. The essential role of Nrf2 in the protection against DN has been reported. Nrf2−/− diabetic mice exhibits higher ROS production and suffer from greater renal injury compared with diabetic mice [14]. The stabilization of endogenous Nrf2 by minocycline offers a protection against Nlrp3-inflammasome-induced diabetic nephropathy [15]. Polydatin promotes the Nrf2 activity to resist the upregulation of FN and ICAM-1 in diabetic mice kidneys [8]. Our previous study showed that connexin43 inhibited the accumulation of ECM and TGF-β1 expression mediated by HG via upregulating the activity of Nrf2 [16].

Astaxanthin (AST) is a fat-soluble xanthophyll carotenoid. This non-toxic and natural dietary carotenoid is a common pigment in algae, shrimp, lobster, crab, salmon, and other organisms [17,18]. The antioxidative potency of AST is reportedly 550-fold greater than vitamin E [19]. AST can improve renal function in diabetic rats and reduce renal cell injury [17,20,21]. In view of the importance of Nrf2/ARE signaling in antioxidant protection, the capability of AST to enhance the resistance to oxidative stress through Nrf2/ARE signaling and then alleviate DN should be explored. Thus, this study aimed to determine whether AST could alleviate the pathological progress of DN by activating Nrf2/ARE signaling and diminishing the excessive oxidative stress and FN accumulation in GMCs challenged with HG.

## 2. Results

### 2.1. Effect of AST on Metabolic Parameters, Renal Morphology and ECM Accumulation in Streptozotocin (STZ)-Induced Diabetic Rats

**Figure 1:**
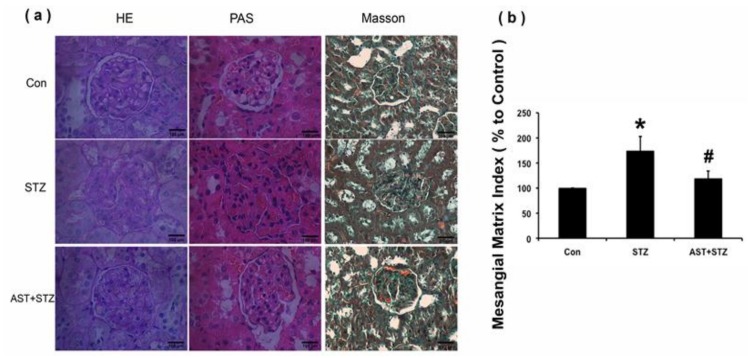
Glomerular injury in STZ-induced diabetic rat kidney. (**a**) Tubulointerstitial fibrosis in the kidneys of the control, diabetic, and AST groups was detected by hematoxylin-eosin (HE) (magnification 400×, Scale bar represents 100 μm) and Masson and (Green indicates collagen; magnification 200×, Scale bar represents 200 μm); (**b**) Glomerular histopathology was assessed by Periodic Acid-Schiff staining. The pictures display the representative glomeruli of periodic-acid Schiff (PAS)-stained sections in the control, diabetic, and AST groups at an original magnification of 400×. Scale bar represents 100 μm. Mesangial matrix index in the PAS-stained glomeruli was semi-quantified as described in Materials and Methods. * *p* < 0.01 vs. control group, ^#^ *p* < 0.05 vs. diabetic group.

As shown in Table 1, kidney weight and kidney hypertrophy index (KW/BW), fasting blood glucose, blood urea nitrogen, serum creatinine, and urine protein over 24 h were significantly increased in STZ-induced diabetic rats compared with those in the control group (*p* < 0.05, Table 1). After 12 weeks of therapy with AST (25 mg/kg daily i.g.), the diabetic rats exhibited a significant reduction in these parameters except blood glucose (*p* < 0.05; Table 1). Figure 1a shows the representative glomerular histology of the hematoxylin-eosin (HE)-stained, Masson-stained, and periodic-acid Schiff (PAS)-stained sections. Compared with the age-matched control rats, STZ-induced diabetic rats displayed a prominent glomerular accumulation of the PAS-positive matrix. The matrix expansion in the AST-treated group was significantly lower than that in the untreated group (Figure 1a). To determine the effect of AST therapy on mesangial matrix accumulation, we evaluated the mesangial matrix index (MMI, in %) and found it to be significantly increased in the diabetic rats compared with the normal group. Treatment with AST markedly reduced the MMI in the glomeruli of diabetic rats (*p* < 0.05, Figure 1b). HE and Masson staining showed that the diabetic rats presented renal damage, including reduced tubular epithelial cells and tubular atrophy. Increased ECM deposition was also observed in the interstitial tissues (Figure 1a).

### 2.2. Effect of AST on the Protein Expression of FN, TGF-β1 and ICAM-1 in the Kidneys of Diabetic Rats

**Figure 2:**
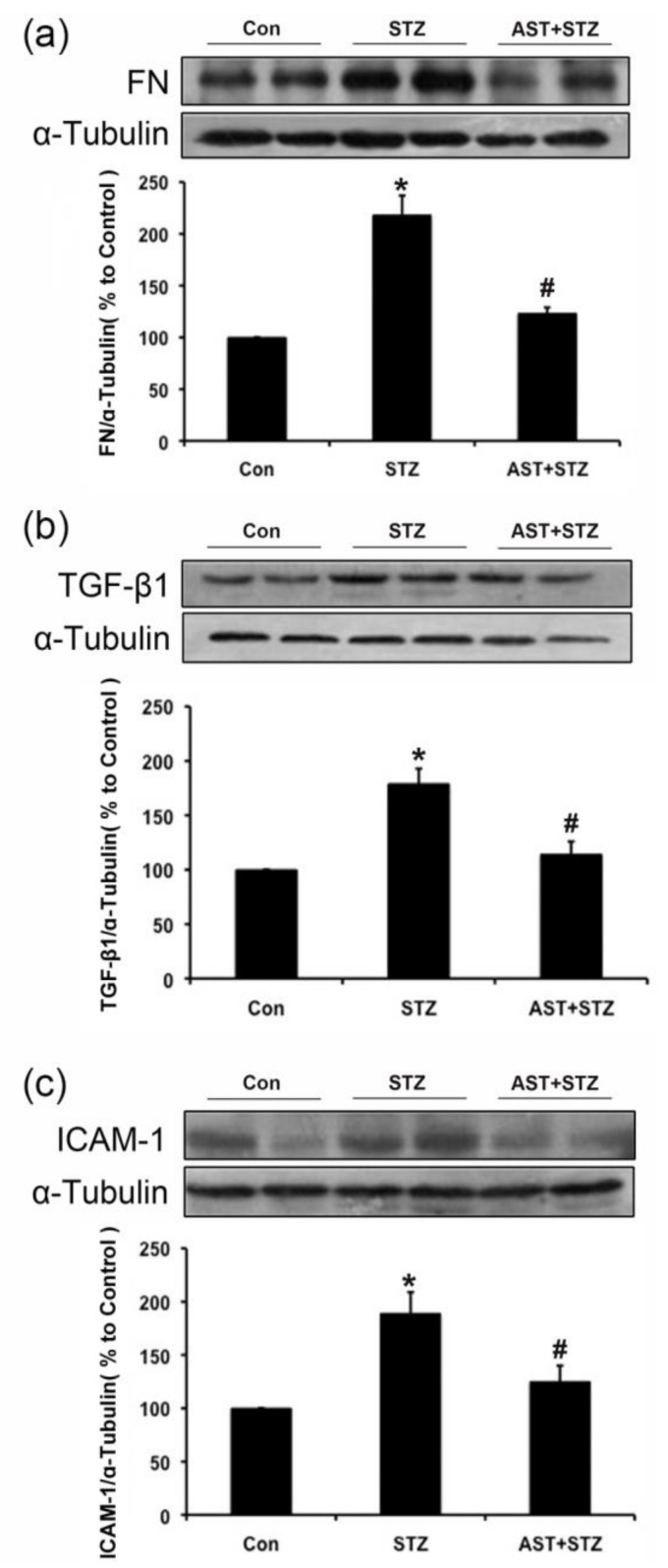
AST inhibited the protein expression of fibronectin (FN), transforming growth factor (TGF)-β1 and intracellular adhesion molecule (ICAM)-1 in the kidneys of diabetic rats, as revealed by Western blotting. (**a**–**c**) After 12 weeks of AST (25 mg/kg daily i.g.) treatment for diabetic rats, we explored the effect of AST on the protein expression of FN, TGF-β1 and ICAM-1 in the kidneys. α-Tubulin was measured as the loading control. All experiments were performed independently at least thrice with similar results. * *p* < 0.05 vs. control group, ^#^ *p* < 0.05 vs. diabetic group.

Hyperglycemia promotes the accumulation of ECM, such as FN, increases the TGF-β1 expression and promotes the overproduction of inflammation factors, including ICAM-1 in diabetes. Thus, we determined whether AST ameliorated the upregulation of the ECM components in diabetic kidneys. In vivo data showed that the protein levels of FN, TGF-β1 and ICAM-1 in the kidneys of diabetic rats were increased compared with those of the control group. After AST treatment, the upregulated FN, TGF-β1 and ICAM-1 protein levels were partially inhibited in STZ-induced diabetic rats (*p* < 0.05, Figure 2a–c). These results, combined with the metabolic parameters and the renal morphology images, confirmed that renal injury was characterized by renal hypertrophy, ECM accumulation, glomerulosclerosis, and renal dysfunction in STZ-induced diabetic rats. However, the overall results indicated that AST could ameliorate the renal injury in STZ-induced diabetic rats.

### 2.3. AST Treatment Prevented the Increased F-Action Formation and Protein Expression of FN, TGF-β1 and ICAM-1 in GMCs Challenged with HG

**Figure 3:**
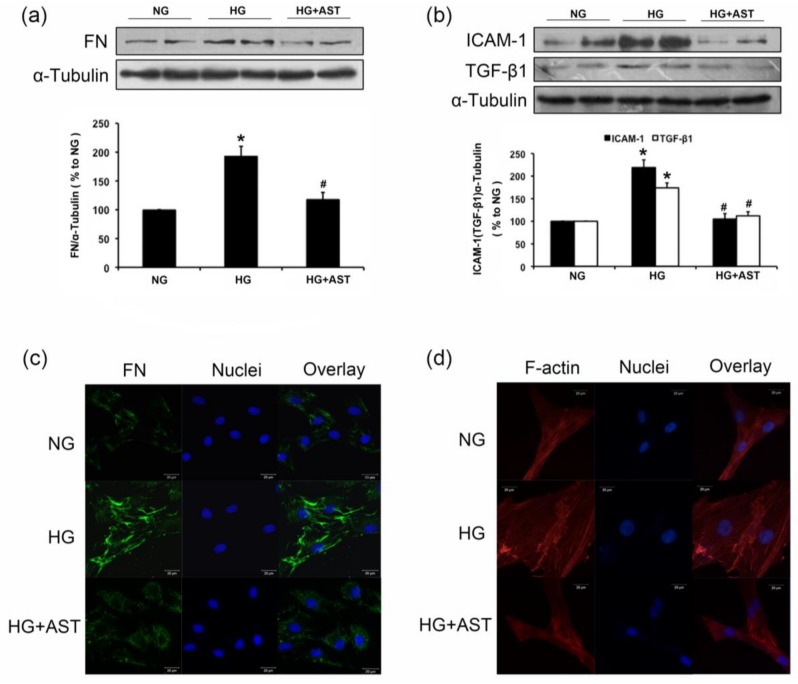
AST inhibited HG induced protein expression of FN, ICAM-1 and TGF-β1 in GMCs, as revealed by Western blotting. (**a**,**b**) After 24 h of AST (10 μM) treatment for glomerular mesangial cells (GMCs) cultured in high glucose (HG), we explored the protein expression of FN, ICAM-1 and TGF-β1. α-Tubulin was measured as the loading control. All experiments were performed independently at least thrice with similar results. * *p* < 0.05 vs. normal glucose group, ^#^ *p* < 0.05 vs. high glucose group. (**c**) shows the immunofluorescence images of FN distribution under the laser scanning confocal microscopy (magnification 400×). Green fluorescence indicates the localization of FN. (**d**) Confocal microscopy was used to evaluate the effect of AST on HG-induced actin cytoskeletal remodeling in GMCs (magnification 630×). Red fluorescence indicates localization of F-actin. Blue fluorescence indicates nuclei. Scale bar represents 20 μm.

Given that ECM accumulation is a pivotal pathologic change in DN, we explored the effect of AST on ECM components in vitro. Similarly, compared with the normal control, the expression levels of FN, TGF-β1 and ICAM-1 significantly were increased after 24 h of HG treatment. However, AST treatment attenuated FN, TGF-β1, and ICAM-1 accumulation in GMCs cultured in HG (*p* < 0.05, Figure 3a–c). In addition, HG enhanced actin stress fiber formation. However, HG-induced actin stress formation was notably reduced in cells co-incubated with AST, suggesting AST played a positive role in HG-induced cytoskeletal remodeling (Figure 3d).

### 2.4. AST Boosted the Activity of SOD and Total Antioxidative Capacity, and Reduced the Malondialdehyde (MDA) Content and H_2_O_2_ Level in GMCs Treated by HG

**Figure 4:**
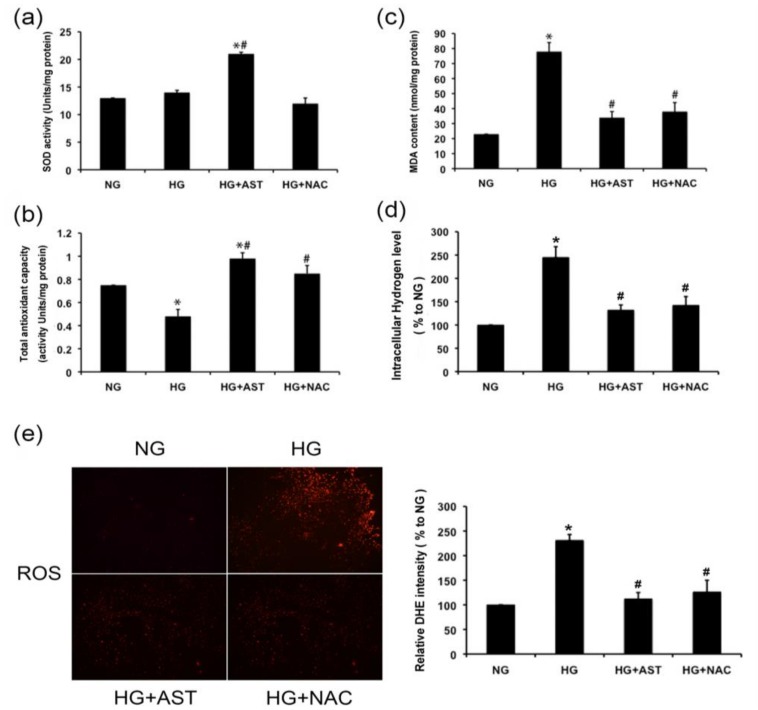
AST enhanced the antioxidant capacity reduced by HG. (**a**) After 12 h of AST (10 μM) treatment for HG-induced GMCs. Superoxide dismutase (SOD) activity was measured and expressed as units/mg protein; (**b**) AST (10 μM) reversed the cellular total antioxidant capacity inhibited by HG treatment for 12 h; (**c**) MDA level was measured using thiobarbituric acid method and expressed as nmol/mg protein after 12 h of AST (10 μM) treatment with HG (10 μM); (**d**) AST restrained the intracellular H_2_O_2_ level promoted by HG treatment for 12 h; (**e**) Fluorescent probe dihydroethidium (DHE) was applied to detect the intracellular reactive oxygen species (ROS) levels after 12 h of HG treatment with or without AST. GMCs were loaded with 10 μm DHE incubated in the dark at 37 °C for 30 min under gentle shaking. High-content screening system (Array Scan VTI 600 plus) was used to measure the fluorescence intensity. Experiments were performed independently at least thrice with similar results. * *p* < 0.05 vs. normal glucose group, ^#^ *p* < 0.05 vs. high glucose group.

One of the most important properties of AST is its potent antioxidant activity. ROS overproduction, which is the core of the pathogenesis of DN, has been thoroughly documented in the glomerular compartment in various animal models and isolated glomerular cells in vitro culture systems. Thus, we explored the antioxidant capacity of AST in HG-induced GMCs to elucidate the mechanisms of the renal protective effect of AST. No significant changes were observed for the SOD activity (Figure 4a). The total antioxidant capacity was reduced (Figure 4b). The MDA content and H_2_O_2_ level increased in the GMCs cultured in HG (Figure 4c,d), indicating that oxidative damage occurred. However, AST treatment notably enhanced the activity of SOD and total antioxidative capacity in HG-treated GMCs (*p* < 0.05, Figure 4a,b). Furthermore, AST treatment reversed the increased MDA content and H_2_O_2_ level induced by HG (*p* < 0.05, Figure 4c,d), as well as ROS level (*p* < 0.05, Figure 4e). These results indicated that AST protected GMCs against oxidative damage induced HG.

### 2.5. Nrf2/ARE Signaling Was Enhanced by AST Treatment in HG-Induced GMCs

Several studies, including our previous work, have shown that Nrf2/ARE signaling adaptively activates under HG ambience [22]. However, this response is still insufficient to quench the ROS overproduction in HG-treated GMCs, leading to further injury of the kidney.

**Figure 5:**
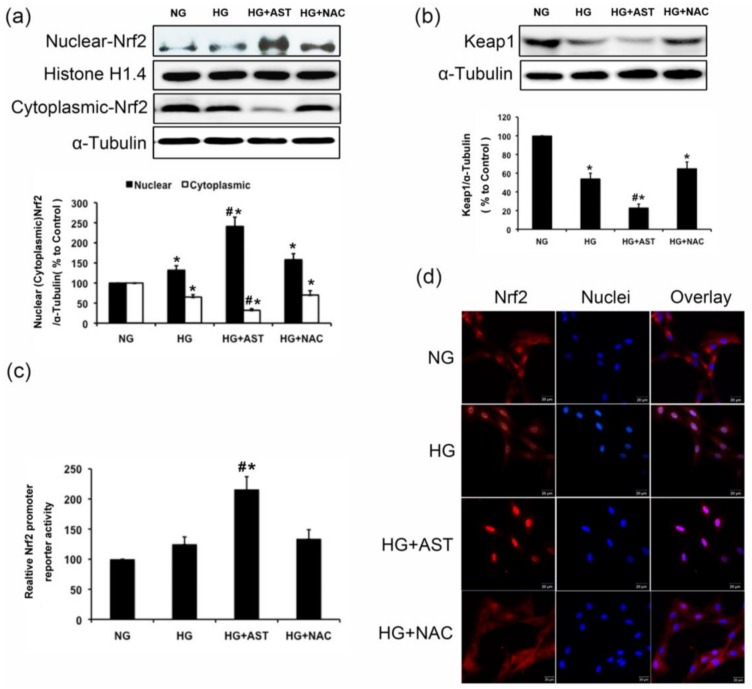
Nrf2/ARE signaling was enhanced by AST treatment in GMCs cultured in HG. (**a**) 1 h of HG treatment induced the nuclear accumulation of Nrf2, and AST treatment enhanced the nuclear accumulation of Nrf2 activated by HG; (**b**) 1 h of AST treatment decreased of Keap1 protein level by HG; (**c**) The effects of AST on the transcriptional activity of Nrf2 were by measured by using luciferase reporter assay after 6 h of HG treatment; (**d**) Immunofluorescence images stained with anti-Nrf2 antibody were captured using a laser scanning confocal microscope (magnification 400×). Red fluorescence indicates Nrf2, and blue fluorescence indicates the nucleus. α-Tubulin was measured as the loading control. Experiments were performed independently at least thrice with similar results. * *p* < 0.05 vs. normal glucose group, ^#^ *p* < 0.05 vs. high glucose group.

Therefore, appropriate intervention is necessary to enhance the activity of Nrf2/ARE signaling in renal cells to protect against kidney damage induced by ROS. Abundantly higher level of Nrf2 expression was found in the cytoplasm than in the nucleus of GMCs under normal conditions (Figure 5a). Treatment with HG for 1 h increased the nuclear content of Nrf2 and decreased the total keap1 protein level in GMCs (*p* < 0.05, Figure 5a,b). The immunofluorescence data was highly consistency with the immunoblotting results (Figure 5d). Luciferase reporter assay was designed to determine the transcriptional activity of Nrf2. A slight increased transcriptional activity of Nrf2 was observed after 6 h of HG treatment, which was further promoted by the AST treatment (*p* < 0.05, Figure 5c). These results suggested that AST quenched the excessive ROS production induced by HG by activating the Nrf2/ARE signaling.

### 2.6. The Protein Expression of SOD1, HO-1 and NQO1 Were Further Enhanced by AST Treatment in GMCs Challenged with HG

**Figure 6:**
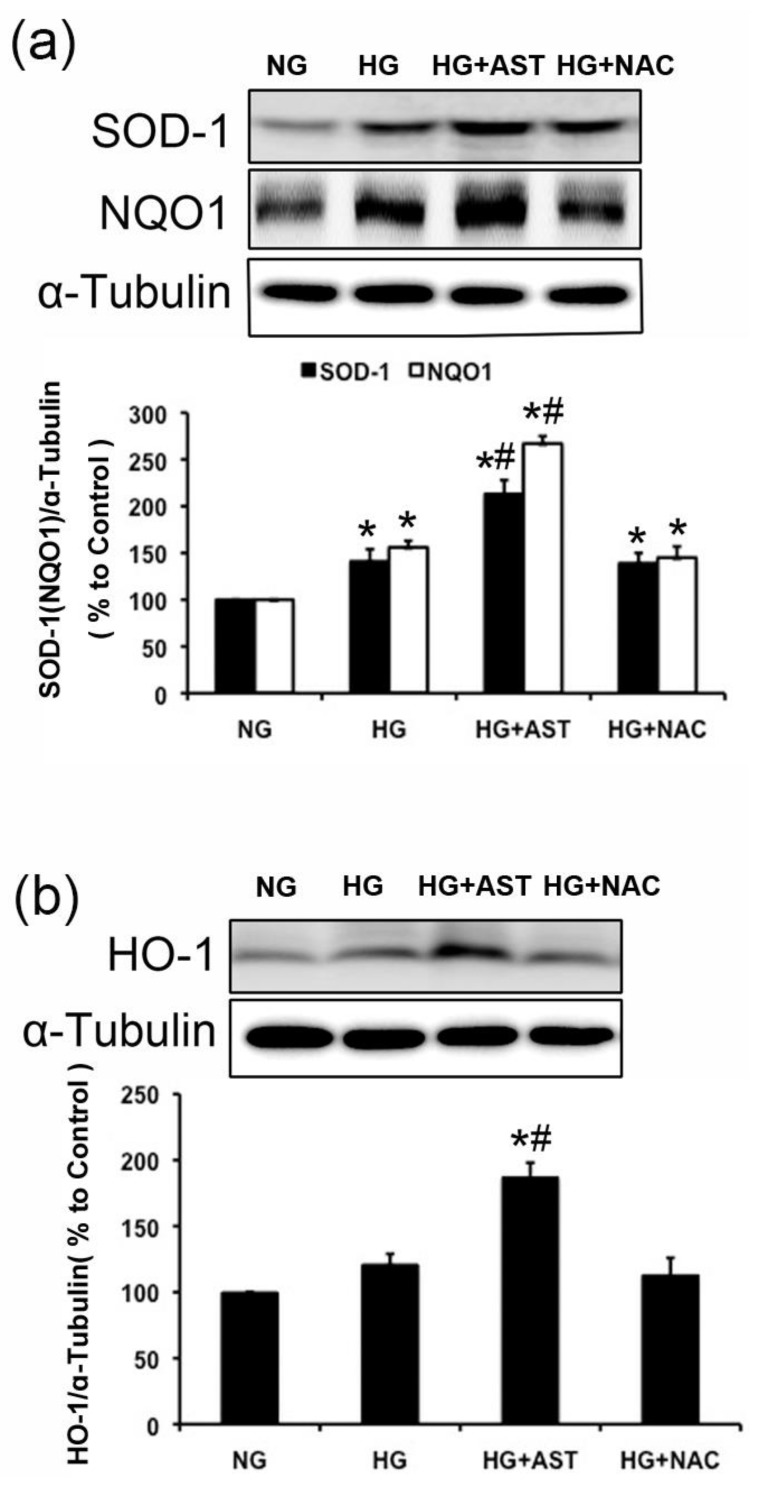
AST upregulated the targets of Nrf2/ARE signaling in GMCs challenged with HG. (**a**) The expression of SOD1 and NQO-1 after 6 h of HG treatment in GMCs cultured with AST; (**b**) The expression of HO-1 after 6 h of HG treatment in GMCs cultured with AST. α-Tubulin was measured as the loading control. Experiments were performed independently at least thrice with similar results. * *p* < 0.05 vs. normal glucose group, ^#^ *p* < 0.05 vs. high glucose group.

Since temporal expression pattern of target genes is a key to determine the activity and function of a transcription factor, we then determined the expression of Nrf2 downstream signaling targets SOD1, HO-1 and NQO1, which exert an antioxidant and antitoxic effect. Consistent with Nrf2 content in nuclei, these targets’ expressions were adaptively higher in HG-treated GMCs compared with the levels detected in GMCs incubated with normal glucose. However, a considerable increase of these targets’ expressions was observed after AST treatment in HG-induced GMCs compared with the HG group (*p* < 0.05, Figure 6a,b). These data suggest that the downstream targets of Nrf2/ARE signaling were highly activated in AST-treated GMSs with stimulation of HG.

### 2.7. Nrf2 Deficiency Abrogated the Inhibition of FN and TGF-β1 Expression of AST, and Decreased the Antioxidant Capacity of AST in GMCs Treated by HG

**Figure 7:**
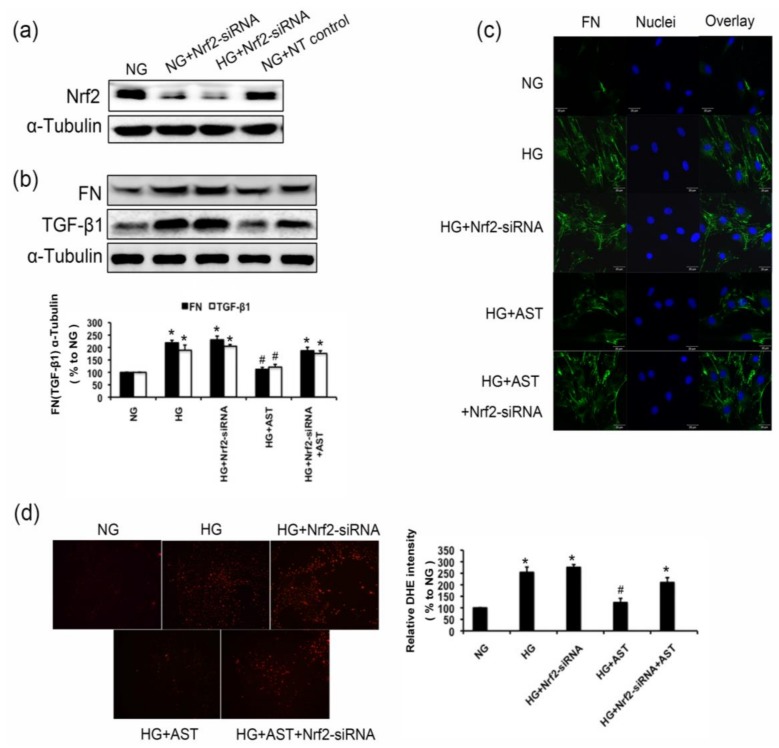
Nrf2 depletion partly abrogated the inhibition of AST on the ROS production and the protein level of FN and TGF-β1. (**a**) GMCs were transfected with small interfering RNA against Nrf2. After transfection for 48 h, the proteins were extracted for analysis of Nrf2 expression by Western blot analysis; (**b**) After 48 h of Nrf2 depletion by small interfering RNA, GMCs were treated by HG with or without AST (10 μM) for 24 h, and the proteins were then extracted for analysis of FN and TGF-β1 expression by Western blot analysis; (**c**) After 48 h of Nrf2 depletion by small interfering RNA, GMCs were treated by HG with or without AST (10 μM) for 24 h. Immunofluorescence images stained with anti-FN antibody were captured using a laser scanning confocal microscope (magnification 400×). Green fluorescence indicates FN, and blue fluorescence indicates the nucleus; (**d**) After 48 h of Nrf2 depletion by small interfering RNA, fluorescent probe DHE was applied to detect the intracellular ROS levels after 12 h of HG treatment with or without AST. GMCs were loaded with 10 μm DHE incubated in the dark at 37 °C for 30 min under gentle shaking. High-content screening system (Array Scan VTI 600 plus) was used to measure the fluorescence intensity. α-Tubulin was measured as the loading control. Experiments were performed independently at least thrice with similar results. * *p* < 0.05 vs. normal glucose group, ^#^ *p* < 0.05 vs. high glucose group. NT, non-targeting control.

We hypothesized that Nrf2-mediated antioxidant defenses play a pivotal role in the protection of AST for GMCs challenged with HG. To confirm this hypothesis, we used loss-of-function analysis on the Nrf2 expression and monitored the oxidant levels and the expression of FN and TGF-β1 in GMCs upon stimulation with HG. Genetic ablation of Nrf2 with the specific siRNA against Nrf2 resulted in >90% depletion of the protein levels of Nrf2 (Figure 7a). The Western blot data and immunofluorescence images showed that the knockdown Nrf2 abrogated the inhibition of FN and TGF-β1 expression in AST-treated GMCs upon the stimulation of HG (*p* < 0.05, Figure 7b,c). Furthermore, after the Nrf2 expression was depleted, AST could not effectively inhibit the ROS level induced by HG (*p* < 0.05, Figure 7d). These results suggested that the protection effect of AST for renal cells depended on the Nrf2/ARE signaling activation.

## 3. Discussion

Several studies have reported that AST positively affected DN. AST improved antioxidant enzymatic activities, significantly reduced the MDA and protein carbonyl levels compared to that of diabetic rats, and attenuated the glomerular hypertrophy and tubular dilatation of diabetic rats [17]. Long-term AST treatment resulted in decreased levels of blood glucose and urinary albumin as well as DNA damage in diabetic mice [20]. Another study reported that AST protected proximal tubular epithelial cells against oxidative stress, inflammation, and apoptosis induced by HG treatment [23]. The proposed mechanism by which AST may prevent and treat diabetic microvascular complications is by modulating oxidative stress, inflammation, and apoptosis by quenching free radicals and preventing the formation of advanced glycation end products [23,24]. However, the specific mechanisms responsible for the renal protective effect of AST in DN remain unclear.

DN is one of the most severe microvascular complications of diabetes mellitus. The most important characteristic of DN is the accumulation of ECM [25,26]. Therefore, terminating the excessive generation of ECM induced by hyperglycemia can prevent and treat DN. ICAM-1 is a major downstream inflammatory factor [27], and an ICAM-1 gene deficiency presents a protective effect against nephropathy in type 2 diabetic db/db mice [28]. TGF-β is closely related to chronic inflammation as well as glomerular and tubular hypertrophy, which promotes the occurrence of oxidative stress [29]. In this study, FN, ICAM-1 and TGF-β1 were induced in kidneys of diabetic rats and in GMCs cultured with HG whereas AST reversed these pathological changes. Excessive actin stress fiber formation is a hallmark of cell hypertrophy. AST also inhibited the actin stress fiber formation in HG-treated GMCs. Morphological analysis revealed that the diabetic rats exhibited excessive accumulation and structural damage of the glomerular mesangial matrix. However, treatment with AST ameliorated these morphological changes, suggesting that AST can interfere with the pathogenesis of DN by reducing the accumulation of ECM components. 

Although the mechanisms responsible for the effects of HG on FN and TGF-β1 expression are complex, the excessive generation of ROS is considered a primary factor in these processes. ROS could induce the generation of growth factors and cytokines, which would enhance renal cell hypertrophy or proliferation by upregulating the ECM expression and decreasing their degradation, ultimately leading to renal fibrosis [30,31]. Our previous studies have verified that treatment with 30 mM HG for 12 h enhanced ROS generation in GMCs. The current study showed that the excessive ROS was markedly quenched by AST treatment, as was the case in another report [21]. *N*-acetyl-l-cysteine (NAC), which is an oxidant used as the positive control, also inhibited the ROS level induced by HG. In addition, AST restored the SOD activity and total antioxidative capacity inhibited by HG. Moreover, AST downregulated the higher MDA content and H_2_O_2_ level in GMCs treated by HG. The antioxidative capacity of AST is due to its unique structure consisting of long conjugated double bonds, which facilitate antioxidant activities by quenching the singlet oxygen and scavenging radicals to terminate chain reactions [32]. However, we speculated that the renal protective effect of AST on DN is partially dependent on its structure. Thus, future studies should elucidate the actual mechanisms.

Oxidative stress associated with diabetes is secondary to increased ROS production and diminished antioxidant capacity [6]. The latter is mainly caused by impaired Nrf2 activation and translocation. The transcription factor regulates the gene encoding antioxidants and molecule detoxification [11,13]. However, in the present study, Nrf2 was slightly increased in the nuclei of GMCs induced by HG and decreased in Keap1, which is its repressor. This finding is consistent with our previous study [22]. Enhanced Nrf2 in response to hyperglycemia was found in both cultured cells and the kidneys of diabetic mice [33]. Slightly increased Nrf2 expression was observed in diabetic mice at two weeks and two months after diabetes onset [34]. Similarly, we also found enhanced Nrf2 levels in the kidneys of diabetic rats. Moreover, the levels of an array of key antioxidant enzymes (SOD-1, HO-1, and NQO1) under the control of Nrf2 were enhanced to a certain level, suggesting that Nrf2 adaptively remained functional to overcome HG damage in GMCs. Unfortunately, the antioxidant capacity and ROS level in HG-treated GMCs in our study suggested that oxidative stress still occurred. Western blot and immunofluorescence analysis revealed increased Nrf2 accumulation in the nuclei after AST treatment in HG-treated GMCs. Antioxidant enzymes under the control of Nrf2, including SOD-1, HO-1 and NQO1 were also upregulated by AST. The HG-induced ROS was effectively quenched in the AST-treated group. Thus, we posited that the antioxidative capacity of AST depends not only on its structure but also on interaction with antioxidative signaling, such as Nrf2/ARE signaling. To confirm this hypothesis, Nrf2 siRNA was used in our study. As expected, after the depletion of Nrf2, the ROS level was not significantly inhibited by AST treatment in GMCs challenged with HG. In addition, AST lost control of the FN and TGF-β1 overexpression induced by HG after Nrf2 depletion. These results verified our hypothesis that AST activated Nrf2/ARE signaling in HG-induced GMCs and enhanced the antioxidative capacity of GMCs to quench ROS to decrease the EMC accumulation in GMCs. However, the mechanism by which AST activates Nrf2/ARE signaling in GMCs remains unclear. AST reportedly accumulates in the cell membrane and mitochondria of human mesangial cells [21]. A recent study showed that the mitochondria-targeted antioxidant MitoQ ameliorated the tubular injury mediated by mitophagy in diabetic kidney disease via Nrf2/PINK1 [35]. In the current study, we observed that AST treatment decreased of Keap1 protein level in high glucose-induced GMCs. It has been reported that many foods (i.e., broccoli, grapes and cinnamon) may contain natural electrophiles that react with Keap1–Cys^151^ and increase Nrf2 levels [36]. A recent study suggests that carbonyl function in fucoxanthin, whose structure is similar to AST, may probably contribute to the oxidization of the silyl group in the Keap1, resulting in the activation of Nrf2 [37]. Because the present of carbonyl group and conjugated double bond in AST structure, it could be an acceptor of electron in Michael addition reaction. We speculate that, as with other natural electrophiles, AST might react with Keap1–Cys^151^ and increase Nrf2 levels via the canonical mechanism in the kidney of diabetic rats. However, this hypothesis needs several well-designed experiments to confirm, which will be our next work.

In the current study, we found that AST treatment alleviated the metabolic parameters, renal morphology and ECM accumulation in STZ-induced diabetic rats. Additionally, HG induced the adaptively activated Nrf2/ARE signaling and increased the expression of FN, ICAM-1 and TGF-β1, as well as the intracellular ROS generation in GMCs. However, AST treatment strongly promoted the nuclear translocation and transcriptional activity of Nrf2 as well as upregulated the expression of SOD1, NQO1 and HO-1, ultimately quenching the higher level of ROS and inhibiting the FN, ICAM-1 and TGF-β1 expression induced by HG.

## 4. Materials and Methods

### 4.1. Reagents and Antibodies

D-Glucose was obtained from AMRESCO (Solon, OH, USA). Bovine serum albumin (BSA, Fraction V) was purchased from Mbchem (Shanghai, China). Penicillin and streptomycin were purchased from Life Technologies (Grand Island, NY, USA). AST was purchased from Sigma (10 μM concentration used in cells experiments, St. Louis, MO, USA). NAC was purchased from Beyotime (Shanghai, China, 4 μM concentration used in cells experiments). Reactive oxygen species assay kit, hydrogen peroxide assay kit, total superoxide dismutase assay kit with WST-8, total antioxidant capacity assay kit with a rapid ABTS method and lipid peroxidation MDA assay kit were obtained from Beyotime (Shanghai, China). pARE-luc was purchased from Beyotime (Shanghai, China). pRL-TK and Dual-Luciferase reporter assay system kit were from Promega (Madison, WI, USA). Antibodies against Nrf2 (1:800, catalogue: sc-30915), FN (1:1000; catalogue: sc-18825), ICAM-1 (1:1000; catalogue: sc-1511) (Santa Cruz Biotechnology, Santa Cruz, CA, USA); TGF-β1 (1:600; catalogue: 3709s) and NQO1 (1:1000; catalogue: 3187) Histone H1.4 (1:2000; catalogue: 41328) (Cell Signaling Technology, Boston, MA, USA); α-Tubulin (1:2000; catalogue: T8203) (Sigma, St. Louis, MO, USA); HO-1 (1:1000; catalogue: A1346) (Abclonal Technology, Woburn, MA, USA); SOD-1 (1:1000; catalogue: 10269-1-AP) (Proteintech Group, Rosemont, IL, USA); Alexa Fluor 488/Alexa Fluor555 (1:1000; Invitrogen, Carlsbad, CA, USA).

### 4.2. Cell Culture and Transfection

Rat GMCs were separated from the glomeruli of Sprague–Dawley (SD) rats and identified via a specific assay as previously described [38]. The cultured cells were used at confluence between the 5th and 8th passages. Confluent cells were rendered quiescent by incubation for 24 h in serum-free medium before treating with glucose (5.6 mM as normal glucose and 30 mM as high glucose) for various times. AST and NAC were added with high glucose (Sigma-Aldrich, St. Louis, MO, USA). Specific siRNA against Nrf2 was designed and synthesized by Invitrogen. The sequence of anti-Nrf2 siRNA oligos were sense 5′-3′: CCGGAGAAUUCCUCCCAAUTT; antisense 5′-3′: AUUGGGAGGAAUUCUCCGGTT. Stealth RNAi^TM^ and siRNA negative control (Invitrogen) was used as the non-targeting control (NT) in our siRNA experiments. Lipofectamine RNA iMAX (Invitrogen) was used for transfection with siRNA (Life Technologies, Carlsbad, CA, USA). siRNA experiments were performed as per the manufacturer’s instruction for Lipofectamine™LTX&Plus Reagent (Life Technologies, Carlsbad, CA, USA).

### 4.3. Animal Experiment

Male Sprague–Dawley rats (*n* = 50, 200 ± 10 g) were supplied by the Experimental Animal Center, Sun Yat-sen University, Guangzhou, China. All animal procedures conformed to the China Animal Welfare Legislation and were reviewed and approved by the Sun Yat-sen University Committee on Ethics in the Care and Use of Laboratory Animals. (Permit Number: 20150603008; Animal Quality Certificate No.: 0007633). All animals were housed under standard conditions with obtaining food and water freely. After fed with regular diet for 1 week, they were assigned to a diabetic model group (*n* = 34), which was fed with high fat high glucose diet for the following 4 weeks, a normal control group (*n* = 8), which were fed normal diet as well as a vehicle group (*n* = 8). After 4 weeks feeding, diabetic model group rats were given a single intraperitoneal injection STZ on 30 mg/kg, freshly prepared. The normal group and vehicle group rats were injected with an equal volume of citrate buffer. Diabetic rats were accepted by the fasting blood glucose measurement ≥ 11.1 mmol/L after 72 h injection. Randomize diabetic rats into an administration group (*n* = 8) to receive AST (25 mg/kg daily i.g.), and the others diabetic rats and vehicle group rats were received an equal volume of olive oil. Rats were sacrificed after 12 weeks treatment. The blood sample was collected from abdominal vein and serum was obtained by centrifuge at 3000× *g* for 15 min and stored at −80 °C Kidney cortex samples were rapidly excised, frozen in liquid nitrogen quickly and then stored at −80 °C or fixed in 10% neutral buffered formalin.

### 4.4. Biochemical and Morphological Studies

Blood glucose, blood urea nitrogen, serum creatinine, and urine protein were analyzed in Hainan General Hospital. Blood urea nitrogen was tested with Roche UREAL (Roche Ltd., Basel, Switzerland), serum creatinine was measured with Roche CREJ2 (Roche Ltd., Basel, Switzerland), and urine protein was determined with Szybio TP (Shenzhiyuan Biological Technology, Wuhan, China) according to the manufacturers’ instructions. They were then analyzed with the Roche cobas 8000 modular analyzer (Roche Ltd., Basel, Switzerland). The cortex of kidneys were separated and fixed in 10% formaldehyde before embedded in paraffin. Sections 4-μm thick were stained with periodic acid-Schiff (PAS) or stained with hematoxylin and eosin (HE). The cross-section yielding the maximum diameter of the glomerulus was photographed. Glomerular tuft areas were analyzed by Image-Pro Plus. 40 glomeruli were chosen form three slides in each animal randomly. The MMI was calculated as the ratio of the mesangial area to the glomerular area ×100. Masson staining was used to evaluate collagen expression. Sections (4-μm) of paraffin-embedded tissues were fixed in Bouin’s solution for 2 h at 37 °C and rinsed with running tap water for 5–10 min to remove the yellow color. The slides were then stained with celestine blue solution for 2–3 min and washed in distilled water. Subsequently, the slides were stained in Mayer’s hematoxylin working solution for 2–3 min, washed in distilled water and differentiated in 1% acetic acid solution for 2–5 min. The slides were then rinsed with running tap water for 5–10 min and stained in ponceau-acid fuchsin solution for 10–15 min. After washing in distilled water, the slides were stained in phosphomolybdic acid solution for 10 min and 2% light green solution for 5 min, and then mounted with resinous mounting medium.

### 4.5. Western Blot Assay

Western blot analysis was performed with the standard protocol as previously described [39]. Briefly, the nuclear and cytoplasmic proteins of GMCs were extracted using a commercially available assay kit (Active Motif, Carlsbad, CA, USA), and total proteins using RIPA with cocktail. The proteins were separated by 8% sodium dodecyl sulfate-polyacrylamide gel electrophoresis (SDS-PAGE), and transferred to PVDF membrane (Bio-Rad Laboratories, Hercules, CA, USA). After blocking with 5% non-fat dry milk in 0.1% Tween-20/TBS (TBST) for 1 h at room temperature, the blots were incubated overnight at 4 °C with corresponding primary antibodies. The membranes were incubated with the corresponding HRP-conjugated secondary antibodies for 1 h at room temperature. Immunoreactive bands were visualized with a GE ImageQuant LAS4000mini (GE healthcare, Waukesha, WI, USA) or exposure to X-ray films then scanned films using HP LaserJet Professional M1213nf MFP, quantified by densitometry using Gel Doc XR System (Bio-Rad, Hercules, CA, USA), and then analyzed using Quantity One Protein Analysis Software (Bio-Rad, Hercules, CA, USA).

### 4.6. Dual Luciferase Reporter Assay

GMCs were seeded in 96-well culture plates and cotransfected with 0.2 μg pARE luciferase (Beyotime, Haimen, China) and 0.04 μg pRL-TK (Promega, Madison, WI, USA) in the presence of high glucose with or without AST. After treatment, cells were lysed and luciferase activity was determined using the Dual-Luciferase reporter assay system kit (Promega, Madison, WI, USA). Luciferase activity was normalized to the renilla luciferase activity.

### 4.7. Detection of Intracellular Reactive Oxygen Species (ROS)

The fluorescent probe DHE (Beyotime, Haimen, China) was employed to detect the intracellular ROS levels. When the treatment was completed, cells were washed twice with PBS and then loaded with DHE (10 μM) in fresh DMEM medium without serum or other additives for 30 min at 37 °C. The fluorescence was then quantified, and the images were collected with ArrayScan VTI 600 plus (Thermo Scientific, Waltham, MA, USA).

### 4.8. Determination of Hydrogen Peroxide Generation

For the analysis of H_2_O_2_ accumulation, the cells were incubated with 100 μL of H_2_O_2_ assay reagent containing xylenol orange (Beyotime Institute of Biotechnology, Shanghai, China) for 30 min at 37° C, and fluorescence was detected using the Microplate Reader at 560 nm wavelength.

### 4.9. Determination of Intracellular Superoxide Dismutase (SOD) and Lipid Peroxidation

Total SOD activities of the samples were determined using the Total Superoxide Dismutase Assay Kit with WST-8 and the MDA level was measured using thiobarbituric acid method (Beyotime Institute of Biotechnology, Haimen, Jiangsu, China) based on the protocols provided by the manufacturer.

### 4.10. Confocal Laser Scanning Fluorescence Microscopy (LSCM)

Different groups of adherent cells were washed with phosphate-buffer edsaline (PBS), fixed with 4% paraformaldehyde in PBS for 20 min, and permeabilized with 0.1% TritonX-100 for 5 min at room temperature. After further washing, the cells were incubated with rhodamine-phalloidin (Cytoskeleton, Denver, CO, USA) for 30 min and a Hoechst 33342 solution (Sigma–Aldrich, St. Louis, MO, USA) was used to counter stain the nucleus for 10 min at room temperature in a dark room. Other cells were incubated with a mouse polyclonal antibody directed against Nrf2 or FN over night at 4 °C after blocking with 10% goat serum. Then the cells were incubated in the dark at room temperature for 1h with a secondary antibody (Alexa Fluor 488/Alexa Fluor555, Invitrogen, Carlsbad, CA, USA). The nucleus was stained with Hoechst33342 as aforementioned. Cells were placed under alaser scanning confocal microscope LSM710, CarlZeiss, Germany) for observation and image acquisition.

### 4.11. Statistical Analysis

The data were assessed using SPSS 22.0 software. Values were expressed by means ± SDs Statistical analyses of data were performed by one-way ANOVA using post hoc multiple comparisons. *p* < 0.05 was considered to be statistically significant. All the experiments were performed at least three times.

## 5. Conclusions

Overall, AST treatment alleviated the metabolic parameters, renal morphology and ECM accumulation in STZ-induced diabetic rats. Additionally, HG induced the adaptively activated Nrf2/ARE signaling and increased the expression of FN, ICAM-1 and TGF-β1, as well as the intracellular ROS generation in GMCs. However, AST treatment strongly promoted the nuclear translocation and transcriptional activity of Nrf2 as well as upregulating the expression of SOD1, NQO1 and HO-1, ultimately quenching the higher level of ROS and inhibiting the FN, ICAM-1 and TGF-β1 expression induced by HG. Considering that AST offers significant effects on resisting oxidative stress and relieving renal fibrosis in diabetes, we believe that AST is a potentially therapeutic strategy in the treatment of DN. The follow-up studies will further clarify the deeper molecular mechanisms of the regulation of AST on Nrf2/ARE signaling and other targets in DN.

## Figures and Tables

**Table 1:** Effects of astaxanthin (AST) on renal metabolic and biochemical parameters in STZ-induced diabetic rats.

Parameters	Control (*n* = 8)	STZ (*n* = 8)	STZ + AST (*n* = 8)
Body weight (g)	484.67 ± 8.28	218.23 ± 13.98 *	319.23 ± 21.51 *^,^^#^
Kidney weight (g)	2.37 ± 0.09	3.31 ± 0.59 *	2.73 ± 0.33 *
KW/BW (%)	0.47 ± 0.04	1.19 ± 0.11 *	0.75 ± 0.18 *^,^^#^
Blood glucose (mM)	5.09 ± 0.37	23.4 ± 2.61 *	21.7 ± 1.81 *
BUN (mM)	5.49 ± 1.11	15.07 ± 3.10 *	9.23 ± 1.52 ^#^
Cr (μM)	26.51 ± 3.29	44.23 ± 7.21 *	27.82 ± 8.55 ^#^
UP (mg/24 h)	13.34 ± 2.78	97.87 ± 27.23 *	66.23 ± 19.34 *^,^^#^

KW/BW: kidney weight and kidney hypertrophy index, BUN: blood urea nitrogen, Cr: serum creatinine, UP 24 h: urine protein for 24 h, Data were means ± SD, *n* = 8. * *p* < 0.01 vs. control group, ^#^ *p* < 0.05 vs. STZ-diabetic group.
